# Supplementary material for: Csde1 binds transcripts involved in protein homeostasis and controls their expression in an erythroid cell line
Source: Sci Rep. 2018 Feb 8;8:2628. doi: 10.1038/s41598-018-20518-7 (PMC5805679; doi:10.1038/s41598-018-20518-7)
Supplement: Supplementary file 1 — Supplemental information: Figures and Python code [file 41598_2018_20518_MOESM1_ESM.pdf]

Supplemental Information for:

Csde1 binds transcripts involved in protein homeostasis and controls their expression in an erythroid cell line

Kat S Moore[1], Nurcan Yagci[1], Floris van Alphen[2], Nahuel A Paolini[1], Rastislav Horos[3], Ntsiki M Held[4], Riekelt H Houtkooper[4], Emile van den Akker[1], Alexander B Meijer[2,5], Peter A.C. ‘t Hoen[6], Marieke von Lindern[1*]

Supplemental figure S1

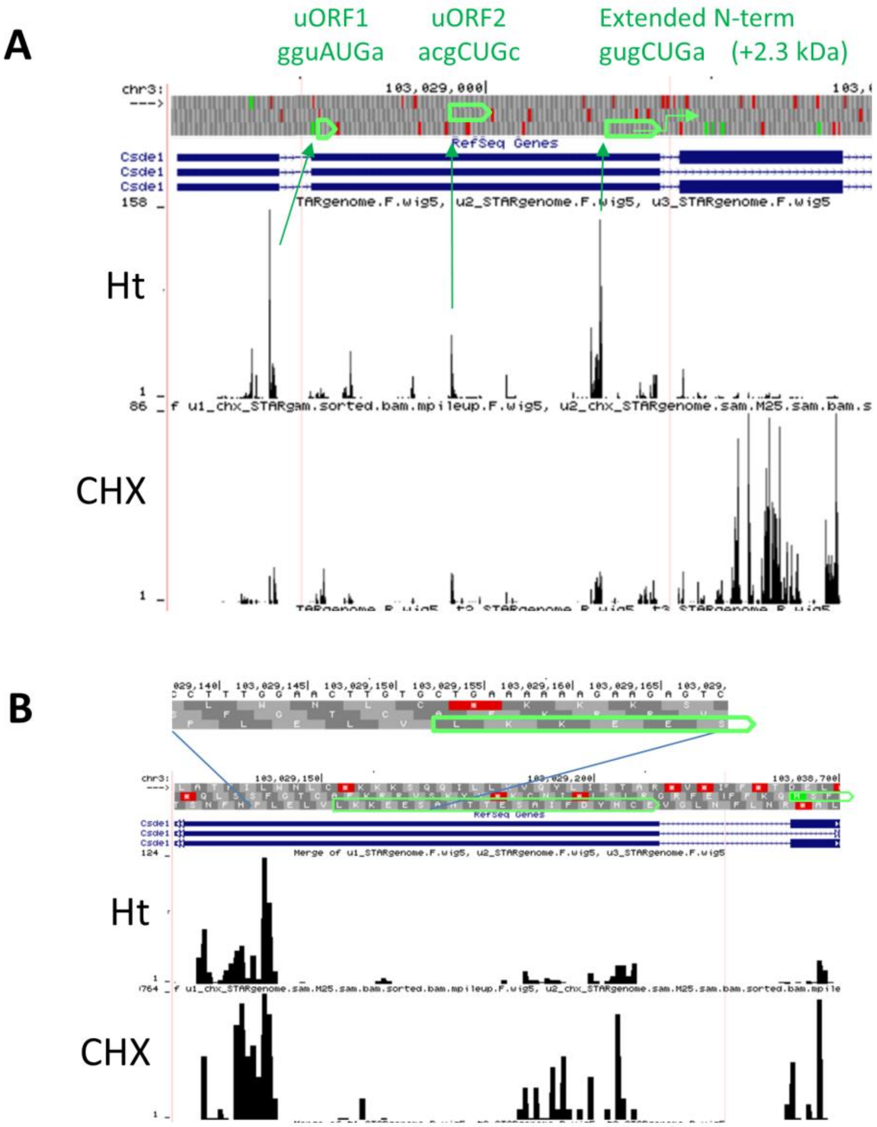

Supplemental figure S1. Ribosome footprinting was performed on erythroblasts (immortalised p53-deficient mouse fetal liver cells [von lindern et al., Oncogene 2001]) using the same protocol used by De Klerk et al [De Klerk et al., Nucl. Acids Res. 2015; manuscript submitted]. Erythroblasts were treated with harringtonin (Ht; 2 µg/ml for 7 min.) to obtain ribosome protected fragments om start codons, or with cycloheximide (CHX; 100 µg/ml for 5 min.) to obtain ribosome protected fragments on elongating ribosomes [Ingolia et al., Cell 2011]. Protected fragments were sequenced and the first nucleotide of the protected fragment was mapped to the UCSC webbrowser. (**A**) Shown are exon 1, 2, and 3 of Csde1 that represent the 5'UTR. The upper track represents Ht reads, the lower track CHX tracks. Green arrows connect peaks in the Ht track to respective uORFs indicated by green arrow shaped squares (uORFs and start codons with relevant Kozak sequence are indicated). (B) Zoom in at the end of exon 2 where a ribosome protected footprint indicates a translation start site at a CUG codon and translation of an N-terminally extended isoform.

Supplemental figure S2

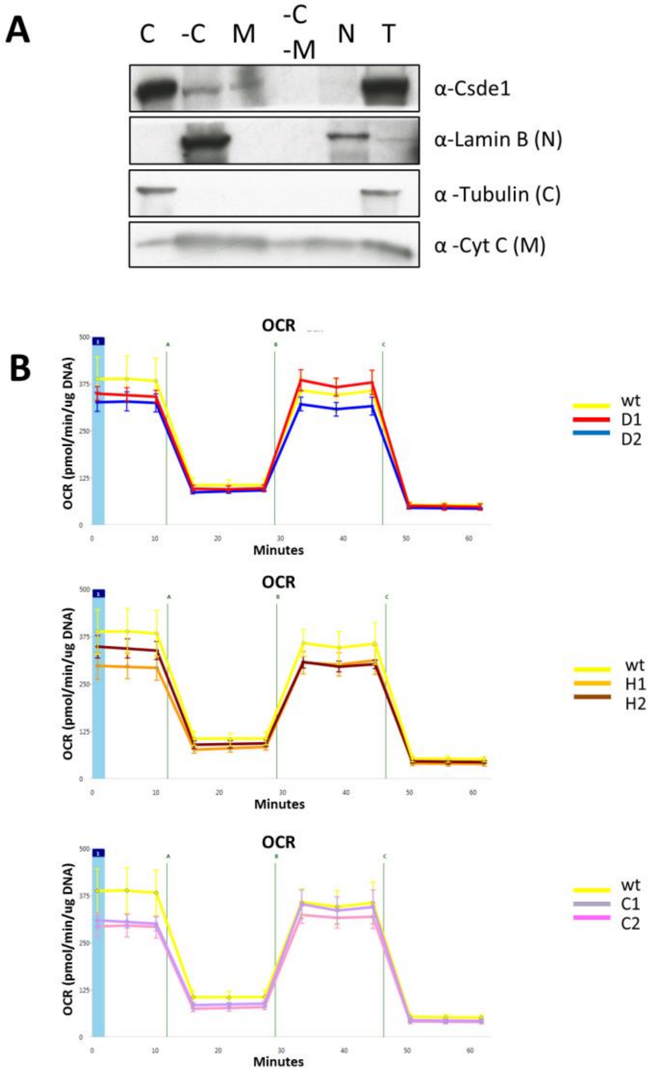

Supplemental figure S2. (A) MEL cell lysate was fractionated in a cytoplamic fraction (C) and the remainder (-C). The remainder was fractionated in a nuclear fraction (N), a mitochondrial fraction (M) and the remainder (-M, -N). Fractions and total cell lysate (T) were assayed on Western blot for Csde1 expression. The Western blot was stained for Lamin B, Tubulin and Cytochrome C as controls for nuclear proteins, cytoplasmic proteins, and mitochondrial proteins, respectively. Depicted are 5 minute exposures for Csde1 and Lamin B1 and 5 second exposures for Tubulin and Cytochrome C. The membrane was cut between regions of interest before staining. The raw images are available as Supplemental Figure S7. **(B)** Oxygen consumption rate (OCR) of Csde1 CRISPR clones versus wild-type, as measured by the Seahorse mitostress kit. OCR is measured in four phases. The first phase represents basal respiration, after which ATP-synthesis inhibitor oligomycin is injected to inhibit mitochondrial respiration. An injection of carbonyl cyanide p-trifluoromethoxy-phenylhydrazone (FCCP), which allows free migration of protons across the mitochondrial membrane, permits a measurement of maximum respiration. In phase 4, an injection of rotenone & antimycin A ends the mitochondrial overdrive state and returns the OCR to minimum activity. Values are corrected for cell input (µg DNA). Error bars represent the standard error of the mean (SEM).

Supplemental figure S3

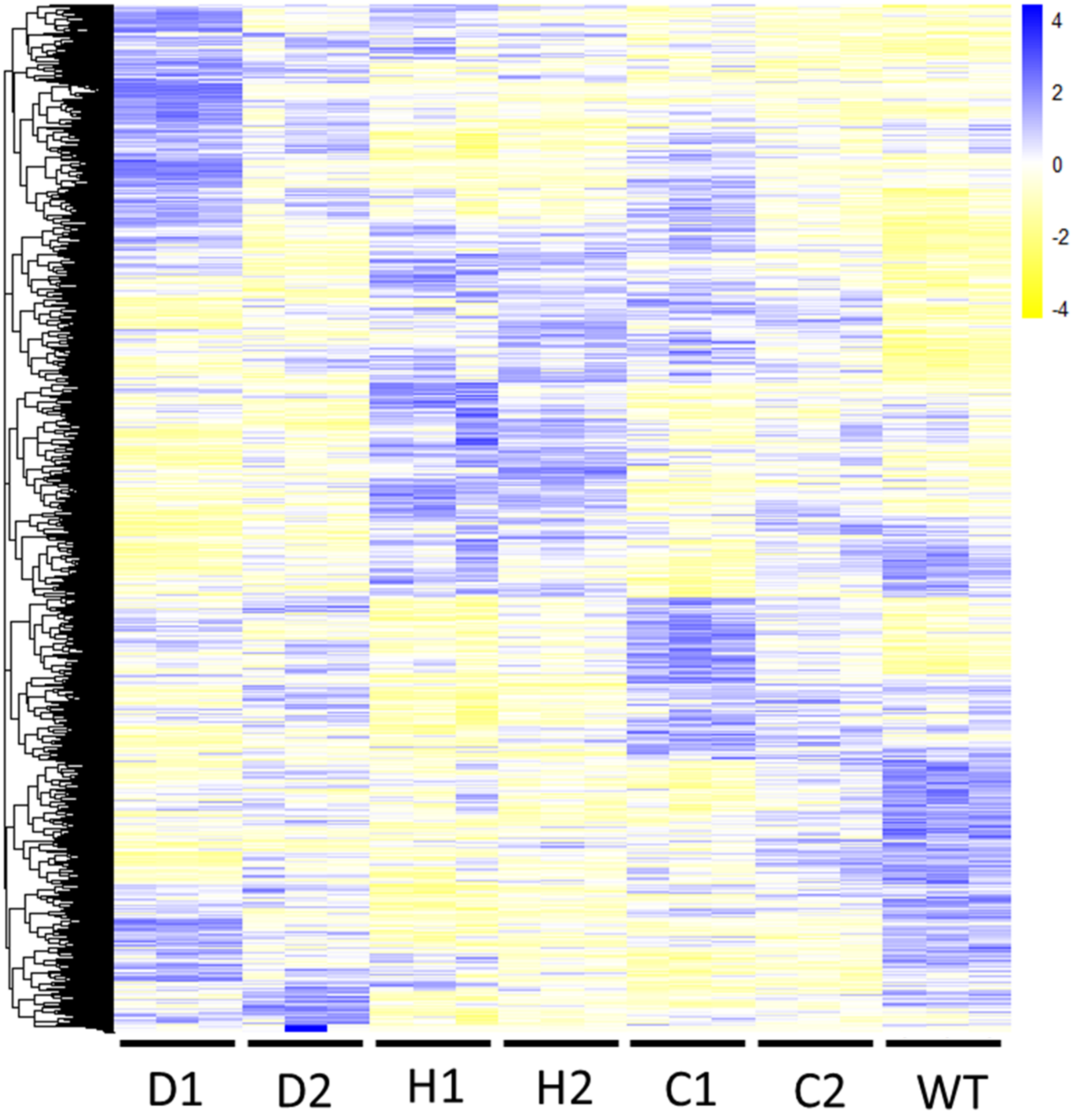

Supplemental figure S3. RNA was isolated from MEL cells. Hm clones (H1, H2), Del clones (D1, D2), control clones (C1, C2) and parental MEL (wt). RNA was sequenced (polyA mRNA), and analysed. Three samples were analysed for each clone. Significant transcripts from ANODEV analysis were subjected to hierarchical clustering. (blue: upregulated, yellow downregulated).

Supplemental figure S4

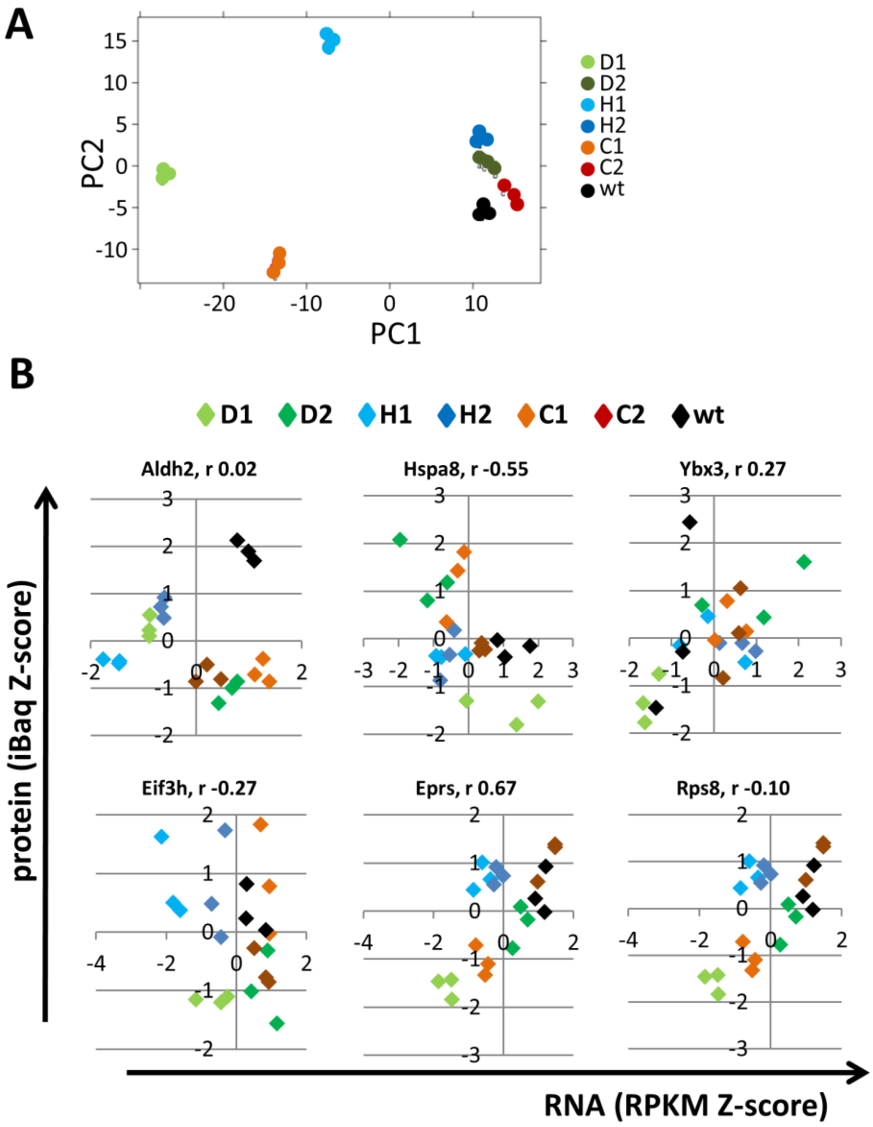

Supplemental figure S4. (A) Principle component analysis of RNA-seq on Csde1 CRISPR clones. (Del clones: green; Hm clones: blue, HET control clones: orange-brown, wtMEL: black). **(B)** Correlation between mRNA and protein expression of select transcripts (Z-scores, standard deviations from the mean). (Del clones: green; Hm clones: blue, HET control clones: orange-brown, wtMEL: black)

Supplemental figure S5

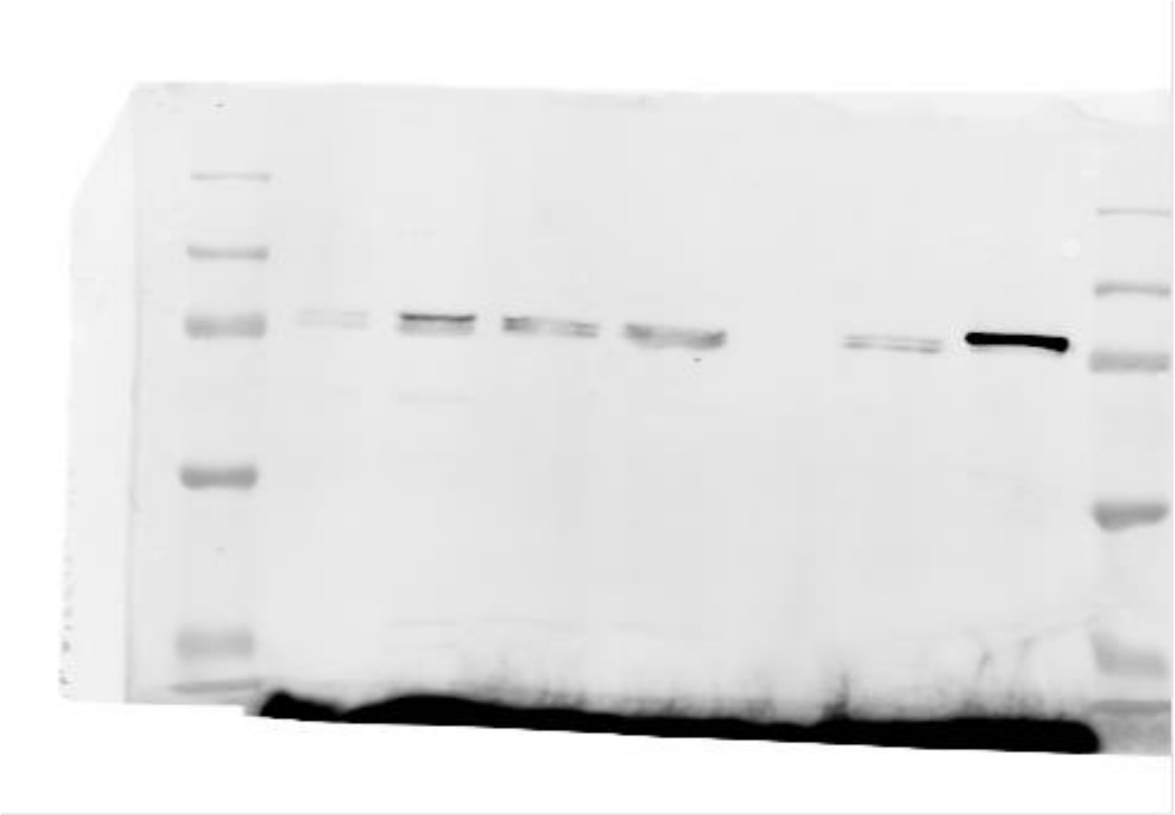

Supplemental figure S5. Uncropped image from Western blot in Figure 1A. Scan was produced using Odyssey software at intensity 5 at 800 nm. Image was exported at black and white.

Supplemental figure S6

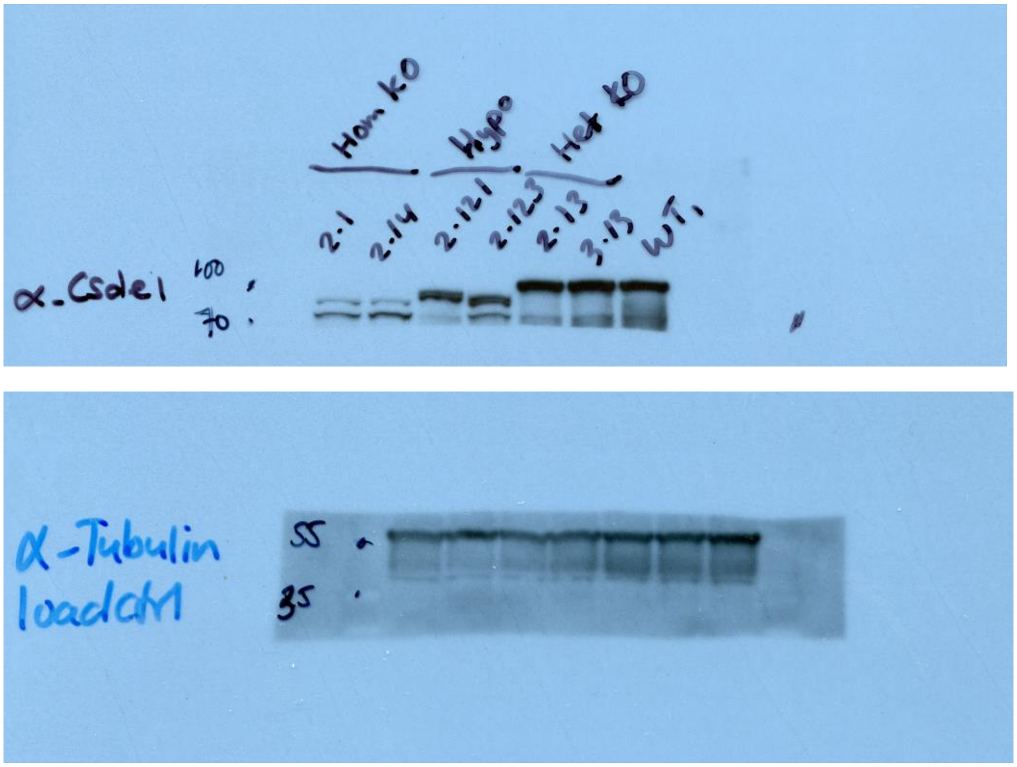

Supplemental figure S6. Uncropped image from Western blot in figure 3C. As per standard Western blot procedure, the membrane was cut to the region of interest and were stained separately using the enhanced chemiluminescence (ECL) kit from Thermofisher on two separate pieces of photo paper. Image produced via a digital scan of photo paper. Both anti-Csde1 and anti-Tubulin are the results of a 15 second exposure.

Supplemental figure S7

A

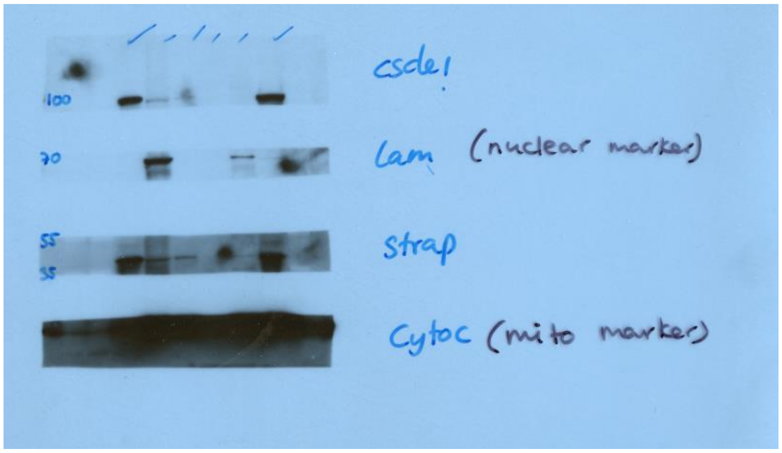

B

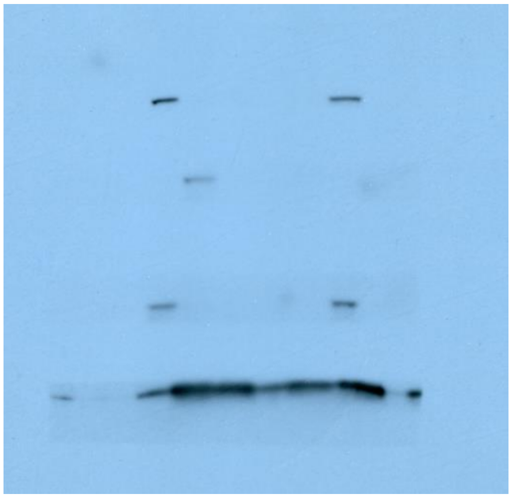

C

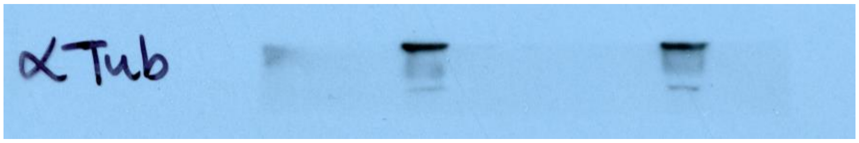

Supplemental figure S7. Uncropped image from Western blot in Supplemental Figure S2A. As per standard Western blot procedure, the membrane was cut to the region of interest and were stained separately using the enhanced chemiluminescence (ECL) kit from Thermofisher. Image produced by digitally scanning photo paper. Strap bands are part of the original image, but do not appear elsewhere in this article. **(A)** Csde1, Lamin B1 and Cytochrome C after a 5 minute exposure. **(B)** Csde1, Lamin B1 and Cytochrome C after a 5 second exposure. **(C)** Tubulin was exposed to a different photo paper for 5 seconds.

Supplemental information: Identification of Csde1 binding sites with Biopython

```
##Before you start:
#You will need biopython installed (http://biopython.org/wiki/Download)
#Create a list of genbank sequences that correspond to the transcripts you want to search
#This can be done by any available method, but one way is
#1) Convert whatever sort of IDs you have to Refseq mRNA IDs (ie. NM_001005419) using (for example)
Biomart from Ensembl
#2) Save those IDs in a text file
#3) Upload that file to batch entrez and search in their nucleotide database
http://www.ncbi.nlm.nih.gov/sites/batchentrez
#4) Under "Display settings", select Genbank-full
#5) Under "Send", select "Complete record" and "send to file"
#6) Start the script using the genbank file as input. Uses Python2.7.6

#This tool is designed to be run for use with an interactive editor such as IEP.
#It can be run as a script, but it is recommended to run each segment (denoted with “##”) sequentially
to see the output and check for errors

##Change working directory, import modules
import os
#Change the next line to reflect your working directory
#os.chdir("/home/kat/Python/Csde1_binding_sites")
print("Working directory is: " + os.getcwd())
import re
from Bio import SeqIO
from Bio import SeqFeature
from Bio.SeqFeature import SeqFeature
from Bio.SeqFeature import FeatureLocation
from Bio.SeqRecord import SeqRecord

##Read the genbank sequences as a dictionary
#Use Bio.SeqIO.parse() to read in sequence data as SeqRecord objects
#The SeqIO.to_dict() function reads the sequences in as a dictionary
#The Refseq ID is then the key, all other info is accessible from value.methods()
#See the Biopython manual for more info

#Change the next line to reflect the name of your .gb file
seq_dict = SeqIO.to_dict(SeqIO.parse("30117_allsig_BIP.gb", "genbank"))
print("Importing " + str(len(seq_dict.keys())) + " sequences.")

##Create a testing dictionary for assertion statements
#Has 2 "type 1" and 2 "type 2" Csde1 binding sites
has_binding_sites =
SeqRecord("TTGGGGGAAGTAATTTGGGGGGGAAACGAGTTTTTTTTTGGGGGAAGTAATTTTTGGGGGGGAAA
CGAGTT", id = "has_binding_sites", description = "Binding site transcript (yes), variant 5")
```

```
fakeCDS = FeatureLocation(2,10)
has_binding_sites.features.append(SeqFeature(fakeCDS, type = "CDS"))

#Has no binding sites
no_binding_sites = SeqRecord("GGGGGGGGGGGGGGGGGGGGGGGGGGGGGGGGGG", id =
"no_binding_sites", description = "No binding sites (nope), variant 2")
fakeCDS = FeatureLocation(3,9)
no_binding_sites.features.append(SeqFeature(fakeCDS, type = "CDS"))

test_list = [has_binding_sites, no_binding_sites]
testing = SeqIO.to_dict(test_list)

##Extract gene name from value.description
def find_gene_name(dictionary, key):
    """Extract the gene name from the description of a seqrecord object.\nUtilizes a regex which finds all
parentheses-enclosed words followed by a comma.\nUse on a dictionary of seqrecord objects created
with SeqIO.to_dict()."""
    value = dictionary[str(key)]
    m = re.search("\(([a-zA-Z0-9]*)\),", value.description)
    gene_name = m.group() #.group() extracts the matched sequence from match object "m"
    gene_name = gene_name.replace("(", "")
    gene_name = gene_name.replace(")", "")
    gene_name = gene_name.replace(",", "")
    return gene_name

assert find_gene_name(testing, "has_binding_sites") == "yes" #Should pass without error
assert find_gene_name(testing, "no_binding_sites") == "nope"

##Presence of potential binding sites
def binding_sites_present(dictionary, key, site_1 = r"[GA]{5}AAGTA[AG]", site_2 =
r"[AG]{7}[AG]AAC[AG]{3}"):
    """Searches for two regular expressions and returns "yes" if either is found and "no" if neither is
found.\nDefault values for "site_1" and "site_2" match the Csde1 binding sites, but other regexs may be
provided by the user.\nUse on a dictionary of seqrecord objects created with SeqIO.to_dict()."""
    value = dictionary[str(key)]
    if re.search(site_1, str(value.seq)) or \
    re.search(site_2, str(value.seq)):
        foundsite = "yes"
    else:
        foundsite = "no"
    return foundsite

assert binding_sites_present(testing, "has_binding_sites") == "yes"
assert binding_sites_present(testing, "no_binding_sites") == "no"

##Store matched binding sites
def binding_site_sequence(dictionary, key, site_1 = r"[GA]{5}AAGTA[AG]", site_2 =
r"[AG]{7}[AG]AAC[AG]{3}"):
```

```
    """Stores the sequence(s) of the recognized patterns as a list.\nDefault values for "site_1" and "site_2" match the Csde1 binding sites, but other regexs may be provided by the user.\nUse on a dictionary of seqrecord objects created with SeqIO.to_dict()."""
    value = dictionary[str(key)] #Extracts the corresponding value per key
    runs1 = re.findall(site_1, str(value.seq)) #Stores the sequence of the matched site
    runs2 = re.findall(site_2, str(value.seq)) #Same for site 2
    allruns = runs1 + runs2
    return allruns

assert binding_site_sequence(testing, "has_binding_sites") == ['GGGGGAAGTAA', 'GGGGGAAGTAA', 'GGGGGGGAAACGAG', 'GGGGGGGAAACGAG']
assert binding_site_sequence(testing, "no_binding_sites") == []

##Locations of binding sites
def binding_site_locations(dictionary, key, site_1 = r"[GA]{5}AAGTA[AG]", site_2 = r"[AG]{7}[AG]AAC[AG]{3}"):
    """Stores the start and stop location(s) of the recognized patterns as a list of tuples.\nDefault values for "site_1" and "site_2" match the Csde1 binding sites, but other regexs may be provided by the user.\nUse on a dictionary of seqrecord objects created with SeqIO.to_dict()."""

    run_positions=[] #Create empty list to hold location tuples
    value = dictionary[str(key)] #Extract values per key
    locations1 = re.finditer(site_1, str(value.seq)) #Finds iterable match objects
    for match in locations1:
        run_start1 = match.start() + 1 #Compensate for python's counting at 0, extract start
        run_end1 = match.end() #Extract end
        runtuple = (run_start1, run_end1)
        run_positions.append(runtuple)

    locations2 = re.finditer(site_2, str(value.seq))
    for match in locations2:
        run_start2 = match.start() + 1
        run_end2 = match.end()
        runtuple2 = (run_start2, run_end2)
        run_positions.append(runtuple2)

    return run_positions

assert binding_site_locations(testing, "has_binding_sites") == [(3, 13), (40, 50), (17, 30), (56, 69)]
assert binding_site_locations(testing, "no_binding_sites") == []

##Extracting the CDS locations
#Using biopythons lists of features within SeqRecord objects

def cds_finder(dictionary, key):
    """This function takes the location of the CDS (when defined), converts it to a string,\ngets rid of ambiguous characters, and returns a simple list with the start of the CDS in\nposition 0 and the end of the CDS in position 1."""
```

```
    value = dictionary[str(key)]
    for feature in value.features:
        if feature.type == "CDS":
            cds = str(feature.location)
            cds = cds.replace("(+)", "")
            cds = cds.replace("[", "")
            cds = cds.replace("]", "")
            cds_list = cds.split(":")
            cds_list = [int(cds_list[0]), int(cds_list[1])]
            return cds_list

assert cds_finder(testing,"has_binding_sites") == [2, 10]
assert cds_finder(testing, "no_binding_sites") == [3, 9]

##Binding site in CDS?
def utr_vs_cds(dictionary, key):
    """Determines whether the binding site falls in the 3'UTR, the CDS, the 5'UTR or spans the junction
between them.\nReturns a list of integers corresponding to how many patterns were found in each
region:\n5'UTR, 5'UTR-CDS-spanning, CDS, CDS-3'UTR-spanning, and 3'UTR, in that order."""
    cds_location = cds_finder(dictionary, key)
    cds_start = cds_location[0]
    cds_end = cds_location[1]

    binding_sites = binding_site_locations(dictionary, key)

    UTR_5_bound = 0
    span_5UTR_CDS = 0
    CDS_bound = 0
    span_CDS_3UTR = 0
    UTR_3_bound = 0

    for i in range(0, len(binding_sites)):
        site = binding_sites[i]
        site_start = site[0]
        site_end = site[1]
        if site_start < cds_start and site_end < cds_start:
            UTR_5_bound = UTR_5_bound + 1
        elif site_start < cds_start and site_end > cds_start:
            span_5UTR_CDS = span_5UTR_CDS + 1
        elif site_start < cds_end and site_end > cds_end:
            span_CDS_3UTR = span_CDS_3UTR + 1
        elif site_start > cds_end:
            UTR_3_bound = UTR_3_bound + 1
        else:
            CDS_bound = CDS_bound + 1

    locations_5_span5_CDS_span3_3 = [UTR_5_bound, span_5UTR_CDS, CDS_bound, span_CDS_3UTR,
UTR_3_bound]
```

```
    return locations_5_span5_CDS_span3_3


##Fix the punctuation
def punctuation_fixer(funky):
    """Fixes the format of the output table by converting lists or tuples to a string and removing the extra punctuation.\nStrips away "[", "]" and single quotes."""
    funky_string = str(funky)
    funky_string = funky_string.replace("[", "")
    funky_string = funky_string.replace("]", "")
    no_more_funk = funky_string.replace("\'", "")
    return no_more_funk

assert punctuation_fixer("Free[ of] 'funk") == "Free of funk"

##Create dictionary with tupled values containing gene name, description, transcript length, match count, binding site sequences and positions
#Uses functions defined above, see comments there for detailed explanation
csde1 = {}
metastat_foundsite = 0
metastat_nosite = 0
metastat_5UTRbound = 0
metastat_3UTRbound = 0
metastat_CDSbound = 0
metastat_5UTR_CDS_spanner = 0
metastat_CDS_3UTR_spanner = 0
spans_5UTR_CDS = 0
spans_CDS_3UTR = 0

for key, value in seq_dict.items():
    gene_name = find_gene_name(seq_dict, key)
    seq_length = len(value.seq)
    description = value.description.replace("Mus musculus ", "")
    description = description.replace(", mRNA", "") #Removes M. musculus and mRNA from descriptions
    description = re.sub("\(([a-zA-Z0-9]*)\).", "", description)
    cds_list = cds_finder(seq_dict, key)
    start_codon = cds_list[0]
    stop_codon = cds_list[1]

    if binding_sites_present(seq_dict, key) == "yes":
        allruns = binding_site_sequence(seq_dict, key)
        matchcount = len(allruns)
        runstring = punctuation_fixer(allruns)
        positions = punctuation_fixer(binding_site_locations(seq_dict, key))
        metastat_foundsite = metastat_foundsite + 1
        binding_5_CDS_3 = utr_vs_cds(seq_dict, key)
        found_in_5_UTR = binding_5_CDS_3[0]
        spans_5UTR_CDS = binding_5_CDS_3[1]
```

```
        found_in_CDS = binding_5_CDS_3[2]
        spans_CDS_3UTR = binding_5_CDS_3[3]
        found_in_3_UTR = binding_5_CDS_3[4]
        metastat_5UTRbound = metastat_5UTRbound + found_in_5_UTR
        metastat_3UTRbound = metastat_3UTRbound + found_in_3_UTR
        metastat_CDSbound = metastat_CDSbound + found_in_CDS
        metastat_5UTR_CDS_spanner = metastat_5UTR_CDS_spanner + spans_5UTR_CDS
        metastat_CDS_3UTR_spanner = metastat_CDS_3UTR_spanner + spans_CDS_3UTR

    else:
        matchcount = 0
        runstring = "None"
        positions = "NA"
        metastat_nosite = metastat_nosite + 1
        found_in_5_UTR = 0
        found_in_CDS = 0
        found_in_3_UTR = 0

    #The .title() method capitalizes the first letter of each word, for aesthetic reasons
    entry = (gene_name, description.title(), matchcount, runstring, positions, found_in_5_UTR,
found_in_CDS, found_in_3_UTR, spans_5UTR_CDS, spans_CDS_3UTR, start_codon, stop_codon,
seq_length)

    print(key + " " + str(entry))

    csde1[key] = entry

total_transcripts = metastat_foundsite + metastat_nosite
print("Transcripts scanned: %d" % total_transcripts)
print("Found site in %d transcripts." % metastat_foundsite)
print("No site in %d transcripts." % metastat_nosite)
print("Found %d sites in 5'UTR." % metastat_5UTRbound)
print("Found %d sites in 3'UTR." % metastat_3UTRbound)
print("Found %d sites in CDS." % metastat_CDSbound)
print("Found %d sites spanning 5'UTR and CDS." % metastat_5UTR_CDS_spanner)
print("Found %d sites spanning CDS and 3'UTR." % metastat_CDS_3UTR_spanner)

##Write output
#Change the name of the outfile to whatever you want
outfile = open("30117_allsig_BIP.xls", "w")
header = "Refseq ID" + "\t" + "Gene Name" + "\t" + "Full Name" + "\t" + "Number of Sites" + "\t" + "Site
Sequence" + "\t" + "Site Position" + "\t" + "5' UTR Matches" + "\t" + "CDS Matches" + "\t" + "3' UTR
Matches"  + "\t" "Match Spans 5'UTR-CDS" + "\t" + "Match Spans CDS-3'UTR" + "\t" + "Start Codon" +
"\t" + "Stop Codon" + "\t" + "mRNA Length" +  "\n"

outfile.write("Transcripts scanned: %d \n" % total_transcripts)
outfile.write("Found site in %d transcripts. \n" % metastat_foundsite)
outfile.write("No site in %d transcripts. \n" % metastat_nosite)
```

```
outfile.write("Found %d sites in 5'UTR. \n" % metastat_5UTRbound)
outfile.write("Found %d sites in 3'UTR. \n" % metastat_3UTRbound)
outfile.write("Found %d sites in CDS. \n" % metastat_CDSbound)
outfile.write("Found %d sites spanning 5'UTR and CDS. \n" % metastat_5UTR_CDS_spanner)
outfile.write("Found %d sites spanning CDS and 3'UTR.\n \n" % metastat_CDS_3UTR_spanner)
outfile.write(header)

for key, value in csde1.items():
    out_row = key
    for i in range(0, (len(value))):
        out_row = out_row + "\t" + str(value[i])
    print("Writing line: " + "\n" + out_row + "\n")
    outfile.write(out_row + "\n")

outfile.close()
```
